# Focused Ultrasound for Sarcomas: A Narrative Review

**DOI:** 10.3390/curroncol32080452

**Published:** 2025-08-12

**Authors:** Nidhi Kuchimanchi, Nicolle Sul, Sai Gajula, Margaret Mercante, Emily Tocco, Mackenzie M. Mayhew, Lynn T. Dengel, Ludimila Cavalcante, Lauren Hadley, Russell Gardner Witt

**Affiliations:** 1 School of Medicine, University of Virginia, Charlottesville, VA 22903, USAszh6bt@uvahealth.org (M.M.);; 2 Focused Ultrasound Foundation, Charlottesville, VA 22903, USAlhadley@fusfoundation.org (L.H.); 3 Department of Surgery, University of Virginia Health System, Charlottesville, VA 22903, USAltd5b@uvahealth.org (L.T.D.); 4 Department of Medicine, University of Virginia Health System, Charlottesville, VA 22903, USA

**Keywords:** focused ultrasound, thermal ablation, sarcoma, MRI-guided HIFU, soft tissue tumor, histotripsy, sonodynamic therapy, hyperthermia, immunomodulation

## Abstract

Sarcomas are tumors of mesenchymal origin that are generally resistant to systemic therapies and prone to local recurrence despite current multimodal treatment approaches. Focused ultrasound (FUS) is a noninvasive therapeutic technology that may enhance standard treatment strategies for primary solid malignancies. FUS exerts its effects through diverse mechanisms, including high-intensity focused ultrasound (HIFU) thermal ablation, histotripsy, sonodynamic therapy, immunomodulation, and hyperthermia-enhanced drug delivery. In this narrative review, we summarize the mechanisms of focused ultrasound that have been investigated for the treatment of sarcomas and highlight the results of preclinical, veterinary, and clinical studies related to this area.

## Simple Summary

Sarcomas are rare cancers that often do not respond well to chemotherapy and may recur even after surgery. Focused ultrasound is a noninvasive technology that uses sound waves to target and treat tumors without cutting into the body. It works through several mechanisms, including heating tumors to kill cancer cells, triggering immune responses, and improving drug delivery. In this narrative review, we explain how focused ultrasound has been studied in laboratory models, veterinary patients, and early clinical trials to treat different types of sarcomas. We also discuss how it could be used alongside surgery or other treatments to improve outcomes. Although more research is needed, focused ultrasound shows promise as a new tool to help manage these difficult-to-treat cancers.

## 1. Introduction

Sarcomas are malignant tumors of mesenchymal origin that can be challenging to treat. The three major challenges in treating sarcomas are (1) limited responsiveness to systemic therapies, necessitating surgical resection as the primary modality; (2) the morbidity of surgery, which is often associated with high rates of local recurrence; and (3) an immunologically “cold” tumor microenvironment, which diminishes the effectiveness of immunotherapy across many sarcoma histologies [1,2,3].

Given these limitations, there is growing interest in novel, less invasive treatment modalities that can enhance tumor control while minimizing morbidity. Focused ultrasound (FUS) is a non-invasive, emerging medical technology that uses focused waves of ultrasound energy to exert therapeutic effects on tissues deep within the body and has recently been recognized as a potential adjunct or alternative to traditional solid tumor therapy. FUS was first applied to human subjects in 1942 when the Fry brothers utilized HIFU to induce small lesions in the brain to treat neurologic disorders, but technical limitations prevented widespread adoption of the technology. In the 1990s, improvements in technology, such as real-time imaging and modern transducer design, led to a resurgence of interest in using FUS to treat tumors [4,5]. There are four mechanisms of action by which focused ultrasound has been studied to treat solid tumors, including sarcomas: thermal ablation, histotripsy, sonodynamic therapy, and hyperthermia [6]. These mechanisms are non-invasive; however, there is a lack of awareness about FUS as a potential treatment modality, and device availability is limited.

In this narrative review, we summarize the mechanisms of focused ultrasound (FUS) relevant to sarcomas with metastatic potential, review current preclinical and clinical evidence across multiple sarcoma subtypes, and discuss how FUS may serve as a noninvasive adjunct to surgery within multidisciplinary care. We highlight the potential for FUS to enhance local tumor control, reduce surgical morbidity, and expand treatment options for patients with inoperable or recurrent disease.

## 2. Methods

**Figure 1:**
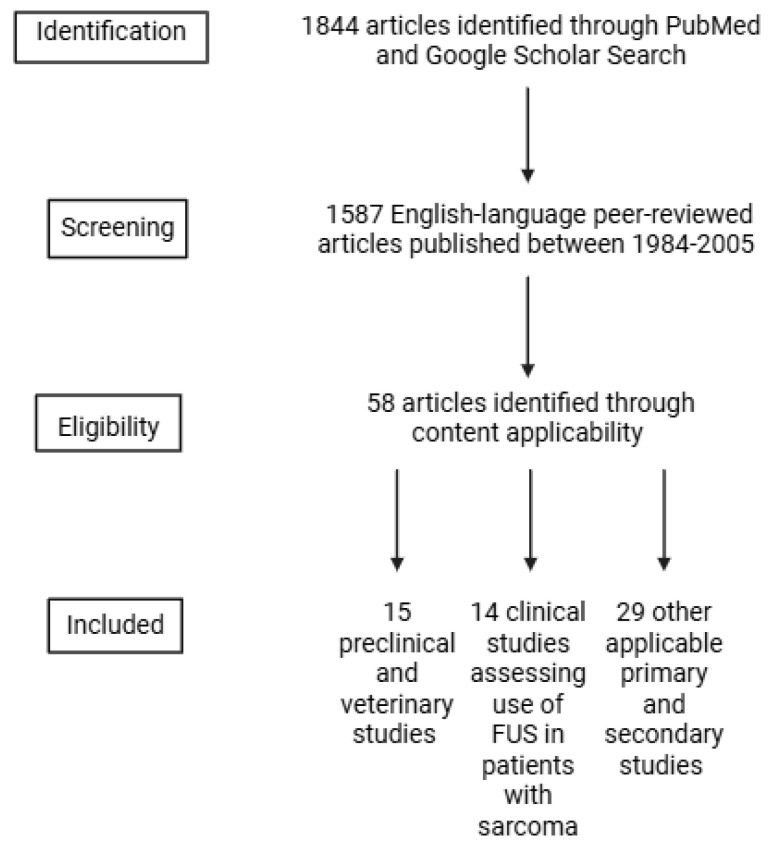
Flowchart diagram outlining study inclusion criteria.

A literature search was conducted in PubMed and Google Scholar using the keywords “focused ultrasound”, “high intensity focused ultrasound (HIFU)”, “histotripsy”, “sonodynamic therapy (SDT)”, “hyperthermia”, and Boolean operations, “and”/”or”, in conjunction with “sarcoma”. The inclusion criteria were (1) peer-reviewed English-language articles, (2) articles published between 1984 and 2025, (3) available full texts, and (4) articles that described sarcoma treatments, FUS mechanisms, and applications of FUS to sarcomas. In total, 58 references were included in this narrative review, including 15 preclinical and veterinary studies and 14 clinical studies. Clinical studies were primarily case reports and case series (Figure 1). Four authors (NK, NS, LH, and RGW) independently screened the articles for applicability. Figures were created in BioRender, and tables were created in Microsoft Excel.

## 3. Mechanisms of Focused Ultrasound for Sarcoma Treatment

### 3.1. High Intensity Focused Ultrasound (HIFU) Thermal Ablation

HIFU thermal ablation was the first mechanism of focused ultrasound to be described and studied. Thermal ablation involves delivering high-intensity, continuous energy via converging ultrasound waves in an accelerated manner, leading to heat production and elevated tissue temperatures (>60 °C). The surrounding vasculature cannot adequately respond to the rapid increase in tissue temperature to cytotoxic levels, and the targeted cells undergo coagulative necrosis. HIFU thermal ablation is extremely precise, with a boundary of no more than 50 μm in width between necrotized cells and normal tissue [4]. HIFU thermal ablation is an image-guided technique and can be guided by either MRI (MRI-HIFU or MRgFUS) or ultrasound, allowing for a spatial assessment of the tumor and its surrounding structures. With MRI-guidance, real-time thermometry measurements allow for temperature monitoring throughout the procedure, informing the treatment team when the desired temperature is reached within the target tissue. Ultrasound guidance is an inexpensive alternative to MRI that allows for real-time imaging of the target throughout the procedure and can identify tissue changes consistent with ablation [7]. HIFU ablation is approved by the United States Food and Drug Administration for treatment of pediatric osteoid osteomas, prostate cancer, uterine fibroids, essential tremor, tremor-dominant Parkinson’s Disease, and Parkinsonian dyskinesia [8,9,10,11,12].

Contraindications to HIFU ablation for solid tumors include the presence of pathologic fractures along the beam path, <0.5 cm between the tumor and skin, and tumor engrossment of a joint, blood vessel, or nerve [13]. MRI-HIFU cannot be utilized in patients with magnetically incompatible devices and is associated with a high cost. HIFU ablation, like traditional imaging ultrasound, is subject to ultrasound artifacts, such as refraction and shadowing. Additionally, lungs and liver parenchyma obscured by overlying rib bones are difficult to penetrate with focused ultrasound beams. While HIFU has been shown to be effective for recurrent, localized, unresectable sarcomas in case reports, its safety and efficacy have not been confirmed by large-scale clinical trials. Adverse effects of HIFU include bone damage and skin burns, sometimes due to the presence of gas reflecting high-energy sound waves back to the transducer [4]. However, this may be circumvented with low-power cumulative energy [14]. Additional adverse effects noted in sarcoma case reports include fever, localized edema, peripheral nerve damage, ligamentous laxity, epiphysiolysis, and secondary infection [15,16].

### 3.2. Histotripsy

Histotripsy, also guided by real-time imaging, is similar to HIFU in that it leads to tissue ablation via noninvasive ultrasound treatment. However, instead of generating thermal energy, histotripsy employs nonthermal, short, high-amplitude acoustic energy to mechanically fragment tissue through the formation of microbubbles from endogenous tissue gas pockets, subsequently leading to tumor cell death in a precise fashion. Histotripsy is currently under investigation to treat breast, kidney, and prostate tumors and is FDA-approved to treat tumors of the liver [4,17,18].

There are several advantages to histotripsy, including its tissue selectivity, creation of sharp boundaries, avoidance of treatment-induced hemorrhage, and stimulation of potent immune responses [17]. Current limitations of this mode of ultrasound ablation are difficulty targeting organs deep within the body, effectively treating targets that have overlying bowel gas and/or breathing motion, and a theoretically increased risk of metastases by dislodging tumor cells [17]. Histotripsy has yet to be investigated as a therapeutic modality for human sarcomas, but there is growing interest in the medico-scientific community to do so, primarily for the potential to induce an immune response.

### 3.3. Sonodynamic Therapy (SDT)

SDT combines low-intensity focused ultrasound with non-toxic chemicals called sonosensitizers to cause cancer cell death. The sonosensitizers preferentially accumulate in cancer cells, and targeted ultrasound energy interacts with the sonosensitizer to generate reactive oxygen species (ROS) that then kill cancer cells [19,20]. The most well-studied sonosensitizer for SDT is 5-aminolevulinic acid (5-ALA), an amino acid used in fluorescence-guided surgery for brain tumors and photodynamic therapy for precancerous skin lesions. The combination of 5-ALA and low-intensity focused ultrasound for SDT has been shown to be effective in preclinical models of high-grade gliomas, and early-stage clinical trials in glioblastoma patients have recently been completed and are awaiting publication (NCT05362409, NCT04845919), while additional clinical trials are ongoing (NCT04559685, NCT06039709). Other sonosensitizers that have been investigated include hematoporphyrin, cyclophosphamide, epirubicin, and docetaxel [21,22,23,24,25]. SDT has been preclinically investigated for the treatment of breast, lung, gastric, liver, ovarian, and prostate cancers, pancreatic ductal adenocarcinoma, and sarcoma [20,26].

The advantages of SDT include sparing of the surrounding normal tissue due to tumor specificity of the sonosensitizers to cancer cells, ability to deeply penetrate tissues, and low systemic toxicity. While the results produced by sonodynamic therapy are promising, disadvantages include a lack of clinically focused ultrasound devices designed to target soft tissue tumors and a paucity of published data on SDT in sarcoma therapy.

### 3.4. Hyperthermia

Hyperthermia is the last FUS mechanism that has been investigated as a therapy for sarcoma. With this approach, high-intensity focused ultrasound is used to temporarily elevate tissue temperature to sub-ablative levels (39–45 °C). The rise in temperature is then used to trigger the local release of the drug from thermosensitive drug liposomes (TSLs) without damaging tissue [27]. Promising results combining mild ultrasound hyperthermia with lyso-thermosensitive liposomal doxorubicin (LTSL-DOX) have led to enhanced drug delivery and antitumor effects in preclinical studies, which led to a clinical trial investigating the safety and efficacy of this approach for pediatric solid tumors (NCT02536183).

The advantages of hyperthermia combined with temperature-sensitive liposome formulations center around the safe, targeted delivery of drug cargo, limiting systemic toxicity. Current challenges include the lack of clinically available thermosensitive liposomes and the need for prolonged ultrasound exposure periods [28].

## 4. Preclinical and Veterinary Studies

Preclinical and veterinary studies have investigated the effects of several focused ultrasound mechanisms of action on sarcoma models and naturally occurring canine and feline soft tissue sarcomas and canine osteosarcoma. Published data are outlined below and summarized in Table 1.

### 4.1. S180 Sarcoma Cell Line

The sarcoma 180 (S180) cell line, characterized by its tumorigenic and invasive nature, was derived from a murine axillary sarcoma over 100 years ago and has been used in over 5000 studies since. The S180 cell line is thought to represent an undifferentiated mesenchymal sarcoma, a high-grade sarcoma with poor prognosis [44,45]. S180 is widely used in preclinical research for studies of tumorigenicity, metastasis, and anti-tumor drug efficacy, owing to its robust growth in multiple inbred mouse strains and its capacity to induce lethal tumors in immunocompetent hosts. For this reason, it has been used in multiple studies assessing the efficacy of FUS [45].

HIFU ablation was tested in a single study of subcutaneous S180 tumors in a mouse model. S180 tumors in 25 mice treated with a single HIFU session were significantly smaller at 14, 21, and 28 days after treatment compared to the control group. Additionally, the HIFU group had a significantly higher survival rate (67%) at 8 weeks compared to that of the control group (23%). Intratumor infiltration by macrophages, helper T-lymphocytes, and cytotoxic T-lymphocytes was also observed in the treatment group, indicating that HIFU ablation induced a tumor-specific immunologic effect. A notable limitation included the use of a single ablative treatment, resulting in only partial coagulative necrosis and incomplete tumor ablation [30].

Sonodynamic therapy has also been studied using in vitro S180 cells. Three different sonosensitizers were tested in conjunction with ultrasound: protoporphyrin IX (PPIX), hematoporphyrin (HPD), and sinoporphyrin sodium (DVDMS) [29,31,32]. The results of these in vitro studies indicate that sonodynamic therapy leads to a decrease in cell viability while increasing the cell damage and apoptosis rate when compared to treatments with no sensitizer (no PPIX: 24.24% ± 5.27%) or ultrasound (0%). The treatment effect was further quantified through the analysis of the concentration of free fatty acids (FFA), antioxidant enzymes, and O2 free radicals (ROS). In particular, the concentration of FFA and ROS increased in the cell membrane using PPIX and in the mitochondria using HPD [29,31]. The use of DVDMS led to no change in the viability of normal cells and no noticeable side effects, suggesting its ability to selectively target tumor cells [32].

### 4.2. Rhabdomyosarcoma and Leiomyosarcoma

HIFU-induced hyperthermia (HT) in combination with thermosensitive liposomes (TSLs) has been investigated in several rodent rhabdomyosarcoma models.

The intratumoral distribution of lyso-thermosensitive liposomal doxorubicin after the administration of HIFU-mediated HT was investigated in 12 rats with subcutaneous rhabdomyosarcoma of the hind legs [35]. Intravenous injection of ^111^In-labeled temperature-sensitive liposomes containing doxorubicin and [Gd] contrast in combination with HIFU hyperthermia targeted at the tumors was performed. Intratumoral doxorubicin concentration was found to be greater in the treatment group vs. controls (4.21 ± 1.41 treated, 1.99 control %ID/g after 48 h). In addition to improved delivery within the tumors, a higher concentration of liposomes (≥50%) remained in circulation for a greater duration in the HT-treated tumors when compared to controls, suggesting that HIFU hyperthermia could further enhance the accumulation of doxorubicin through increased circulation time [35].

Another study involving an engineered murine model of rhabdomyosarcoma (M25FV24C) reached a similar conclusion related to the use of HT to improve the delivery of doxorubicin (n = 65) [34]. Doxorubicin-loaded TSLs were intravenously injected into the mouse, and either 10 or 20 min of HT treatment was applied. There was a significantly greater concentration of doxorubicin within the tumors after 20 min of treatment when compared to 10 min of treatment (3.698 vs. 2.065%ID/g of tumor). These results support the use of HIFU HT in combination with TSLs for greater distribution and accumulation of the drug cargo [34].

An additional study in a rat model of rhabdomyosarcoma investigated a combination of HIFU heating strategies with doxorubicin TSLs to determine an optimal FUS combination therapy for potential rhabdomyosarcoma treatment [36]. Four treatment groups were studied in a total of 113 rats: (1) HT plus dox TSL (continuous wave ultrasound with an average temperature of 41 °C, frequency of 1.44 MHz, and acoustic power of 10–15 W), (2) thermal ablation plus dox TSL (thermal dose, greater than 240 cumulative equivalent minutes at 43 °C; continuous-wave ultrasound with frequency of 1.44 MHz and acoustic power of 35 W), (3) HT followed by thermal ablation plus dox TSL, and (4) no HIFU treatment, just dox TSL. The use of ablation after injection of dox TSL created doxorubicin release along the rim of the tumor and resulted in an increase in drug uptake by the tumor over time. The use of HT resulted in doxorubicin distribution across the tumor in a more homogenous manner (P = 0.036 and 0.016 compared to no hyperthermia groups). All HIFU-treated tumors had delayed growth compared to non-HIFU-treated tumors. HT followed by ablation led to the highest concentration of homogenous drug release across the tumor and had the largest effect on tumor growth over time compared to the other treatment groups. The authors concluded that this was due to direct thermal necrosis of the tumor core and efficient delivery of the doxorubicin to the tumor rim [36].

HIFU ablation was successful in a xenograft nude mouse model of leiomyosarcoma [33]. A total of 65 nude mice were inoculated with ELT-5B uterine leiomyosarcoma cells, with 30 mice growing tumors. The 30 mice that grew tumors were randomly assigned to one of three groups: (1) HIFU treatment (n = 7), (2) sham treatment (n = 7), and (3) control (n = 6). Within three weeks, the HIFU ablation group had a 100% reduction in tumor volume in all but one animal, while sham and control mice saw similar 500% increases in tumor volume. No metastases were observed in HIFU-treated mice over a 3-month period, and histological analysis confirmed complete tumor resolution [33].

### 4.3. Osteosarcoma and Soft Tissue Sarcomas

Canines and felines with naturally occurring sarcomas, specifically osteosarcoma and soft tissue sarcomas (STS), have been treated with focused ultrasound in companion animal clinical trials. A feasibility trial performed by [39] revealed that, among a cohort of 53 dogs with STS, 43 (81%) had STS lesions that were targetable by MRgFUS, and also that most of the targetable lesions (72.1%) could be over 50% ablated. The primary reason for inability to target was complete obstruction by bone or air and/or proximity to the spinal cord (<1 cm) [37]. Following the encouraging results of this feasibility study, a clinical trial investigating the safety, feasibility, and efficacy of MRgFUS partial ablation for the treatment of canine solid tumors was carried out at the Virginia-Maryland College of Veterinary Medicine. A total of 20 dogs were treated, and tumor types included soft tissue sarcoma (n = 15), mast cell tumor (n = 3), osteosarcoma (n = 1), and thyroid carcinoma (n = 1) [38]. HIFU was well tolerated in all but one subject in this study, and histologic examination of treated tumors confirmed partial ablation of each lesion. Immunohistochemical analysis also confirmed the infiltration of T-cell populations at the border between treated and untreated tissue [38].

Another study investigating MRgFUS for both canine and feline STS was performed around the same time as the above trial at Cyprus University of Technology [39]. In this trial, six dogs and four cats with superficial STS were treated with MRgFUS prior to surgical tumor resection. Histopathology confirmed coagulative necrosis within 80% of the STS treated, further confirming the feasibility of MRgFUS for the treatment of veterinary STS and potentially advancing the knowledge of FUS thermal ablation for human STS [39].

In addition to thermal ablation, histotripsy has been studied in clinical trials of canine and feline STS. A trial of three cats with injection site soft tissue sarcoma received histotripsy treatment 3 to 6 days prior to surgical resection. Histological examination confirmed successful ablation of the tumors, with two of three tumor margins clear of sarcoma cells. The outcomes were followed for a median of 358 days, and the cat whose excised tumor had margin involvement experienced STS recurrence, requiring euthanization [41]. The same authors investigated histotripsy to treat naturally occurring STS in canines (n = 10) in a prospective, single-arm, open-label study [40]. After tumor excision, 4–6 days post-histotripsy, histologic examination confirmed successful histotripsy ablation in all 10 tumors. Eight dogs (80%) had their tumors completely excised, while the other two had extensive tumor invasion of the surrounding healthy tissue. After a median follow-up of 112 days, six dogs were alive, two dogs were euthanized due to progressive disease, one dog was euthanized due to an unrelated immune-related thrombocytopenia, and one dog was lost to follow-up [40]. In both trials of histotripsy for veterinary STS, immunohistochemical and gene expression analyses revealed pro-inflammatory changes in the tumor microenvironment, suggesting that histotripsy can stimulate an anti-tumor immune response.

Two additional treat-and-resect clinical trials from the same veterinary group confirmed the feasibility of histotripsy for canine osteosarcoma (OS) [42,43]. In the first study, the safety and feasibility of histotripsy of 5 canine osteosarcoma tumors was confirmed [42]. Spherical ablation volumes between 1.25 and 3 cm in diameter were treated with a single cycle of histotripsy pulses, at 500 pulses per point. Tumor ablation was successfully identified within the target zone in each of the 5 OS cases, although partial ablation was seen within the target zone in some cases. No significant adverse effects were noted [42]. The following study then investigated the safety and efficacy of histotripsy in a portion of 9 canine OS and 1 canine chondrosarcoma [43]. In this study, the number of pulses per point increased from 500 (in the previous study) to 1000, thereby increasing the histotripsy dose, to avoid partial ablation as seen previously. The results echoed the initial study but also concluded that a higher dose leads to increased tissue destruction. This was also the first study where definitive radiographic changes within the ablative zone were noted on post-treatment CT scans [43].

## 5. Clinical Studies

Clinical case reports and series detail the use of HIFU ablation for treatment of a variety of sarcomas, including osteosarcoma, uterine sarcoma, myxofibrosarcoma, spindle cell sarcoma, synovial sarcoma, pediatric sarcoma, and others. Although focused ultrasound has also been investigated for desmoid tumors, these lesions are not included in this section, as our focus is limited to malignant sarcomas with metastatic potential. Details of the included studies are outlined below and summarized in Table 2.

### 5.1. Osteosarcoma

HIFU ablation was first shown to be effective in treating osteosarcomas in 2009 [48]. In a prospective trial of 7 patients with osteosarcomas of the extremities, disease response, pain, and survival were assessed. The inclusion criteria included patients with histopathologically proven extremity osteosarcoma and Enneking IIB disease without neurovascular involvement. Patients with subcutaneous disease involvement and pathological fractures were excluded. The age range was 9–60 years old, and tumor locations included the proximal (n = 1) and distal humerus (n = 2), distal femur (n = 3), and proximal tibia (n = 1). Patients were treated with neoadjuvant high-dose methotrexate/vincristine and doxorubicin/cisplatin therapy for 4–6 weeks prior to HIFU treatment and 2–4 weeks after HIFU treatment. All patients reported elimination of pain without the need for analgesics as well as improved range of motion. Three patients achieved complete response, 3 patients achieved partial response, and 1 patient had progressive disease despite HIFU treatment. The median overall survival time was 68 months. Four years after treatment, 1 patient passed away from cardiac disease, and another passed from cachexia and infection. At five-year follow-up, the overall survival rate was 71.4% (5/7 patients). After five years, 3 patients ultimately passed away from pulmonary metastases. The authors concluded that HIFU ablation may be a limb-salvaging therapy, but larger clinical trials are necessary to evaluate this claim [48].

Subsequent studies showed HIFU to successfully and safely ablate primary malignant bone tumors and relieve malignancy-associated pain [49]. A follow-up to the 2009 study showed that 13 patients with primary bone tumors (12 osteosarcomas and 1 malignant fibrous histiocytoma) had a significant reduction in pain after treatment with HIFU ablation. The overall survival rates at 1-, 2-, 3-, and 5-years after treatment were 100.0%, 84.6%, 69.2%, and 38.5%, respectively. The median overall survival was 43 months [49]. Long-term responses to ablation were not specified.

Another prospective study assessed HIFU ablation outcomes in 80 patients with primary typical osteosarcoma (n = 62), periosteal osteosarcoma (n = 1), periosteal sarcoma (n = 1), chondrosarcoma (n = 10), Ewing sarcoma (n = 3), malignant giant cell bone tumor (n = 1), and unknown histologic type (n = 2) [15]. Patients with osteosarcoma and Ewing sarcoma were also treated with 3–5 cycles of neoadjuvant chemotherapy prior to and 4–6 cycles of adjuvant chemotherapy (cisplatin, doxorubicin, methotrexate, and ifosfamide) after HIFU treatment. Tumors that were not responsive to chemotherapy were treated with HIFU ablation alone. In total, 54 patients achieved complete response after one HIFU ablation session, while another 15 patients achieved complete response after multiple sessions. Among these 69 patients, 7% had tumor recurrence after a mean follow-up of 36.8 months. Overall survival rates at 1-, 2-, 3-, 4-, and 5-years following HIFU ablative treatment for all 80 patients were 89.8%, 72.3%, 60.5%, 50.5%, and 50.5%, respectively. Survival rates between patients with Enneking stage IIb (60 patients) and stage III disease (20 patients) were significantly different. Survival rates at 1-, 2-, 3-, 4-, and 5-years for patients with stage IIb disease were 93.3%, 82.4%, 75.0%, 63.7%, and 63.7%, respectively. Survival rates at 1-, 2-, 3-, 4-, and 5-years for patients with stage III disease were 79.2%, 42.2%, 21.1%, 15.8%, and 15.8%, respectively. Survival rates were significantly greater for patients with stage IIb disease who completed their combined chemotherapy regimens (30 patients) compared to those who did not (30 patients, *p* < 0.001). The authors postulated that HIFU could be added to combination therapies for soft tissue sarcomas soon with continued research efforts [15].

Most recently, HIFU demonstrated effective treatment of local, unresectable recurrence of osteosarcoma [13]. Across 27 patients, the response rate was 51.8% after 2–3 HIFU sessions. Additionally, the median local disease progression-free time was 15 months for patients without lung metastasis and 6 months for patients with lung metastasis. However, 14 of the 21 patients without pulmonary metastasis developed pulmonary metastasis on later follow-up. The authors suggest that this may indicate the need for systemic therapy in conjunction with HIFU [13].

### 5.2. Uterine Sarcoma and Leiomyoma

A retrospective analysis of 15,759 patients with presumed uterine fibroids that presented at various hospitals in Sichuan, China, between November 2008 through September 2019 and underwent HIFU ablation found that 17 patients from this cohort were misdiagnosed and were histopathologically confirmed to have uterine sarcoma [5]. In total, 11 of these patients with uterine sarcomas were diagnosed before HIFU treatment, and 6 were diagnosed after treatment. There were no significant differences between histological type, margin of lesions, or enhancement of lesions on MRI that could explain the cause of misdiagnosis. After an average follow-up of 29.3 months, 10 patients remained living, and 1 patient was lost to follow-up out of the 11 patients diagnosed with uterine sarcoma prior to receiving HIFU ablation. Of the 6 patients diagnosed with uterine sarcoma after HIFU treatment, only 1 patient reported abnormal vaginal bleeding on follow-up. After a median follow-up period of 20.5 months, 1 patient was lost to follow-up, and 4 patients remained living. One patient passed away from their sarcoma 39 months after HIFU treatment [5].

### 5.3. Myxofibrosarcoma

A single case report by Zhao et al., 2021, detailed the first use of HIFU to treat a poorly controlled recurrent myxofibrosarcoma [54]. The patient underwent five cycles of low-power HIFU with complete ablation of the tumor. No tumor relapse was noted on serial MRIs during a 30-month follow-up period. Additionally, the patient did not experience any complications from HIFU treatment, which the authors attribute to their use of low-power cumulative HIFU. The authors suggest that HIFU ablation therapy could be a safe and effective method to treat recurrent, local myxofibrosarcoma with support from future clinical studies [54].

### 5.4. Spindle Cell Sarcoma

There are 2 case reports detailing the use of HIFU in the treatment of spindle cell sarcoma (SCS) [14]. In the first report, a 69-year-old female patient with recurrent SCS in the left thigh, despite undergoing multiple therapeutic surgeries, was treated with 5 HIFU sessions to the location of the recurrence. The authors reported successful total ablation of the lesion, suggesting that HIFU is an effective non-invasive treatment for recurrent SCS. However, it is important to note that the patient experienced bone damage that required surgical correction after HIFU treatment [14]. Additional clinical studies with a greater number of patients and longer follow-up periods are required to determine the safety and efficacy of HIFU for SCS.

In the second report, a 51-year-old male with spindle cell sarcoma of the left chest wall had previously undergone four cycles of chemotherapy with local recurrence. The patient was subsequently treated with 5 cycles of HIFU ablation, and residual tumor cells were not found on repeat biopsy. The authors reported complete ablation of the tumor without complications or the need for additional chemotherapy, radiotherapy, or immunotherapy. No long-term outcomes were reported [51].

### 5.5. Pediatric Sarcomas

HIFU offers an enticing alternative treatment approach for pediatric sarcomas. Although there are no published clinical trial results, a 2016 study evaluated the feasibility of MRgFUS to treat pediatric sarcomas (n = 121) [52]. The investigators found that approximately 64% of patients with pediatric sarcoma had a primary lesion that was targetable by MR-HIFU at diagnosis. The majority of such tumors were osteosarcomas and Ewing sarcomas, making up 31% and 21% of targetable tumors, respectively. In contrast, only 14% of metastatic tumors were targetable. Additionally, 35% of patients with relapsed disease had at least one targetable tumor. The authors comment that the location of the tumor determined targetability, and extremity and pelvic tumors were more likely to be targetable than elsewhere in the body. Most metastases at diagnosis (79%) and lesions in recurrent disease (66%) were in the chest, which were not targetable due to difficulties in ultrasound transmission and respiratory motion [52]. Therefore, while MR-HIFU could pose as a new therapeutic strategy for more than half of newly diagnosed targetable non-metastatic pediatric sarcomas, further studies should focus on improvements in the functionality of HIFU for use in more advanced cases. Two options were suggested to enhance the utility of HIFU: (1) the use of respiratory motion compensation or breath holds, which was shown to increase the number of targetable primary lesions by 9%, and (2) the use of MR-HIFU induced hyperthermia in combination with radiation therapy or chemotherapy [52]. Previous phase III trials, which used other hyperthermia techniques and radiation therapy, have demonstrated better local control in patients with recurrent breast, head and neck, and melanoma tumors [55].

Following the feasibility results above, one clinical trial registered on clinicaltrials.gov was completed (NCT02076906) at Children’s National Hospital in seven pediatric and young adult patients with refractory or relapsed solid tumors, including multiple subtypes of sarcoma. This clinical trial investigated the safety and feasibility of MRgFUS thermal ablation, and the publication of results is pending. An additional clinical trial from the same group at Children’s National Hospital attempted to investigate the safety and feasibility of MRgFUS thermal ablation and hyperthermia combined with lyso-thermosensitive liposomal doxorubicin (ThermoDox) in children and young adults with relapsed/refractory solid tumors (NCT02536183). However, this study was terminated after the enrollment of only two patients due to slow study recruitment secondary to restrictive inclusion/exclusion criteria based upon HIFU device limitations. A similar clinical trial at UT Southwestern (NCT02557854) aimed to investigate HT combined with only liposomal doxorubicin; however, this trial was terminated for an unknown reason.

### 5.6. Other

A single-center retrospective case series attempted to ascertain the efficacy and safety of HIFU treatment in 36 patients with local, unresectable soft tissue sarcoma recurrence [16]. Patients presented between January 2015 through December 2016 and underwent HIFU ablation of their recurrent sarcoma. Pathologic subtypes included lipoblastoma (n = 8), undifferentiated pleomorphic sarcoma (n = 7), fibrosarcoma (n = 6), chondrosarcoma (n = 4), synovial sarcoma (n = 3), leiomyosarcoma (n = 3), aggressive fibromatosis (n = 2), alveolar rhabdomyosarcoma (n = 1), clear cell sarcoma (n = 1), and primitive neurotodermal tumor (n = 1). The primary outcome was local disease progression-free survival (LPFS), and secondary outcomes were 3- and 12-month local tumor response rate, progression-free survival, overall survival, and pain relief. Follow-up imaging 3 months after HIFU treatment showed that no patients achieved a complete response, 47.3% had a partial response, 33.3% had stable disease, and 19.4% had disease progression. Twelve months after HIFU treatment, 38.9% had a partial response, 16.7% had stable disease, and 44.4% had progressed. Median LPFS, progression-free survival, and overall survival were 13 months, 10 months, and 20 months, respectively. In total, 27 patients had disease progression after 12 months; 16 of these patients had metastasis prior to secondary relapse or local recurrence, 9 had metastases after local secondary relapse, and 2 had simultaneous metastases and local secondary relapse. Of the 33 patients who reported pain prior to receiving HIFU treatment, 9 patients achieved complete remission of pain, 16 patients achieved partial remission, and 8 patients reported no improvement in pain. Pain ratings remained stable at 3-month follow-up [16]. HIFU efficacy and adverse effects were not reported by sarcoma subtype, and no long-term outcomes were assessed.

## 6. Ongoing Clinical Trials

There are two ongoing clinical trials investigating focused ultrasound for sarcomas (Table 3). The first is a single-site, single-arm interventional study that aims to evaluate the rate and severity of adverse events from the MRgFUS treatment of newly diagnosed or metastatic undifferentiated pleomorphic sarcomas (NCT04123535). In this trial, 20 adult participants with newly diagnosed or metastatic undifferentiated pleomorphic sarcoma are treated with MRgFUS and compared to an age and sex-matched control group. Secondary objectives include measuring possible immune responses to MRgFUS through serological analyses and flow cytometry.

The second clinical trial is based in the United Kingdom, and the aim is to determine the safety and feasibility of HIFU ablation for resectable and unresectable STS and unresectable small symptomatic intra-abdominal desmoid tumors (NCT05111964). A minimum of 10 patients with malignant STS will be treated with HIFU ablation. The primary objectives are safety, feasibility, and efficacy of HIFU ablation for resectable and unresectable STS and unresectable small symptomatic intra-abdominal desmoid tumors. One-year survival rates, local recurrence, quality of life, and immune response post-ablation will also be evaluated.

While these clinical trials will begin to inform the oncology community of the potential impact of HIFU ablation on sarcoma treatment, additional studies will be needed to optimize HIFU therapy. Ideally, the current clinical trials and published case studies highlighted here will encourage the technical and scientific focused ultrasound communities to design a device that is ideal for sarcoma treatment.

## 7. Focused Ultrasound as a Surgical Adjunct in Sarcoma Care

FUS is a noninvasive therapeutic modality that includes thermal ablation, histotripsy, sonodynamic therapy, and hyperthermia. Across various solid tumors, including sarcomas, these mechanisms have demonstrated promising preclinical and early clinical results. However, the integration of FUS into the multidisciplinary management of sarcoma is still in its infancy, as the clinical applications and efficacy are being determined.

Surgical resection remains the cornerstone of curative treatment for most sarcomas and is typically combined with neoadjuvant radiation and/or chemotherapy to improve resectability and reduce local recurrence [1,2,3]. Yet, surgical morbidity and high rates of local failure, particularly for recurrent or anatomically challenging tumors, underscore the need for adjunctive modalities. In this context, FUS offers a compelling, noninvasive approach to augment surgical strategies, either by enhancing local control in patients with unresectable or recurrent disease or by serving as a palliative tool to alleviate pain and preserve function.

Several case reports and small series have demonstrated the utility of FUS in soft tissue and bone sarcomas, showing tumor regression, improved local control, and pain palliation [13,14,16,51,54]. MRgFUS, in particular, has shown efficacy in alleviating pain caused by bone metastasis [15,48,49,56,57,58]. In a 2022 prospective study, patients with bone metastases were treated with three Tesla-MRgFUS and demonstrated significant reduction in pain, improved functional outcomes, and minimal adverse effects [56]. Health economic modeling has also shown that MRgFUS provides faster pain relief than radiotherapy at a favorable cost-effectiveness ratio [57]. The FURTHER trial, a randomized international study, will help clarify the comparative utility of MRgFUS, external beam radiotherapy, or both for tumor-induced bone pain [58]. These studies underscore the potential role of MRgFUS in sarcoma palliative care, particularly for patients who are not candidates for further surgery or systemic therapy.

Despite encouraging early data, the clinical integration of FUS into sarcoma care remains limited. Existing studies are primarily case reports or small series, often without control groups or long-term outcomes. Comparative trials assessing the efficacy of FUS against or alongside surgery, radiation, or systemic therapy are lacking. Furthermore, FUS implementation is challenged by technical limitations, including tumor proximity to bone, bowel, and neurovascular structures, difficulties in targeting deep lesions, and limited device availability in low-resource settings. Adverse effects such as nerve injury, tissue necrosis, or adjacent tissue damage, though rare, further underscore the need for cautious application and standardized protocols.

From a surgical perspective, FUS may be best positioned as an adjunctive or alternative tool in cases where resection is incomplete, anatomically infeasible, or unlikely to yield durable local control. Its ability to induce targeted tumor destruction without incision could complement surgical decision-making, reduce the need for repeat procedures, and improve quality of life in advanced-stage patients. This approach may also serve as a primary treatment option for patients with indolent, slow-growing disease in whom early surgical intervention may be unnecessary or overly morbid. Further clinical trials should focus on defining specific indications, evaluating safety and efficacy across histologic subtypes, and determining how FUS can be optimally integrated into surgical planning.

Further research is needed to define the clinical utility of FUS and establish its role in sarcoma treatment. Key priorities include (1) prospective, randomized controlled trials comparing FUS to standard-of-care therapies; (2) combination protocols evaluating FUS with systemic chemotherapy, immunotherapy, or radiotherapy; (3) correlative studies investigating FUS-induced changes in the tumor microenvironment and incorporating imaging or molecular biomarkers for treatment monitoring; and (4) subtype-specific investigations to identify histologies most responsive to FUS. These studies will be essential for guiding patient selection and determining how FUS can be most effectively integrated into multidisciplinary sarcoma care.

## 8. Conclusions

Focused ultrasound is emerging as an alternative noninvasive treatment for sarcomas due to its unique ability to interact with tissue and cancer cells via multiple mechanisms of action. Numerous preclinical laboratory and clinical case studies have reported focused ultrasound as a safe and effective therapy for a variety of different sarcoma subtypes. Despite these promising data, few clinical trials are underway to assess the safety and feasibility of focused ultrasound for sarcomas. More clinical trials of focused ultrasound for sarcoma are needed and should concentrate on the safety and efficacy of HIFU thermal ablation, histotripsy, and sonodynamic therapy in this difficult-to-treat group of cancers. Furthermore, improved FUS technology, with features and functionality that are specifically designed to treat tumors of soft tissue and bony origin, could lead to increased interest among the medico-scientific oncology community to investigate FUS mechanisms for the treatment of sarcoma.

## Figures and Tables

**Table 1:** Preclinical Studies.

Reference	Sarcoma Type	Animal Model (n)	Focused Ultrasound Mechanism	FUS Parameters	Findings
[29]	S180	N/A	SDT	30 s, 2.2 MHz, 3 W/cm^2^	Protoporphyrin IX and FUS increased free fatty acid concentration and decreased antioxidant enzyme activity.
[30]	S180	Mouse (*n* = 25)	Thermal ablation	10 s, 3 MHz, 10 W	Using HIFU for partial ablation had an immunological antitumor effect but was insufficient to completely eliminate tumor.
[31]	S180	N/A	SDT	3 min,1.75 MHz,1.4 ± 0.07 W/cm^2^	Apoptosis mechanisms of FUS and of hematoporphyrin differ, which could explain the synergistic effect as treatment.
[32]	S180	N/A	SDT	30/60/90 s, 1.1 MHz, 2 W	Sinoporphyrin sodium and FUS resulted in tumor tissue destruction, cancer cell apoptosis, inhibited angiogenesis, and suppressed cancer cell proliferation.
[33]	Uterine leiomyosarcoma	Mouse (n = 65)	Thermal ablation	2.0 MHz, 2000 W/cm^2^	HIFU treatment completely reduced tumor volume in all mice. No metastasis was observed after 3 months.
[34]	Rhabdomyosarcoma	Mouse (n = 65)	Hyperthermia (HT)	10/20 min, 2.5 MHz	HT increased the intratumoral concentration of doxorubicin. Heating the tumor for 20 min has the most consistent delivery.
[35]	Rhabdomyosarcoma	Rat (n = 12)	Hyperthermia (HT)	20 s, 1.44 MHz, 5–10 W	HT allowed for more homogenous and widespread delivery of radiolabeled liposomes across tumor
[36]	Rhabdomyosarcoma	Rat (n = 113)	Hyperthermia (HT) and ablation	Hyperthermia: 15 min, 1.44 MHz, 10–15 W Ablation: >240 cumulative mins, 1.44 MHz, 35 W	Hyperthermia ensured homogenous drug delivery across tumor when compared to sham and ablation-only groups. The combination of hyperthermia and ablation led to the highest concentration of homogenous delivery.
[37]	STS	Canine (n = 53)	Thermal ablation	N/A	Targetability of most STS in dogs confirmed. Truncal and axillary tumors had the highest targetability, while head and spine had lower targetability.
[38]	STS, mast cell tumor, osteosarcoma, thyroid carcinoma	Canine (n = 20)	Thermal ablation	40–50 s, 5–10 MHz	Thermal ablation increases immune activity in tumor after treatment, particularly in T-cell activation. Thermal damage noted after treatment.
[39]	STS	Canine (n = 6) Feline (n = 4)	Thermal ablation	10/60 s, 2.6 MHz, 50/75 W	Thermal ablation caused coagulative necrosis, but some cancer cells remained intact. FUS should be used with radiotherapy or chemotherapy to eliminate remaining cells.
[40]	STS	Canine (n = 10)	Histotripsy	500 kHz, PNP: 22.60 ± 7.21 MPa	Histotripsy was well tolerated and feasible in canine STS. Pro-inflammatory changes were noted in the TME.
[41]	STS	Feline (n = 3)	Histotripsy	1 MHz, PNP: 29.59 ± 6.08 MPa	Histotripsy was well tolerated and is feasible in feline STS. Pro-inflammatory changes were noted in the TME.
[42]	Osteosarcoma	Canine (n = 5)	Histotripsy	500 kHz, PNP: 29.59 ± 8.17 MPa	Histotripsy is safe and effective for canine osteosarcoma.
[43]	Osteosacoma	Canine (n = 10)	Histotripsy	500 kHz, PNP: 26.57 ± 3.82 MPa	Histotripsy ablation is safe and feasible in lytic or prolieferative canine OS and chondrosarcoma (n = 1), with or without soft tissue extension. More extensive tissue destruction was observed after histotripsy of 1000 PPP compared to 500 PPP. Radiographic changes within the tumor ablation zone were noticeable on post-histotripsy CT scan.

**Table 2:** Clinical Studies.

Reference	Title	Sarcoma Type	Focused Ultrasound Mechanism	N	Findings
[46]	Unsuspected uterine leiomyosarcoma: magnetic resonance imaging findings before and after focused ultrasound surgery	Uterine leiomyosarcoma	Thermal ablation	1	Six months after FUS treatment, tumor decreased in size. However, a circular area of the tumor showed increased intensity on T2-weight imaging.
[47]	Avoiding treatment of leiomyosarcomas: the role of magnetic resonance in focused ultrasound surgery	Uterine leiomyosarcoma	N/A	1	47-year-old female received expedited diagnosis because of MRI screening that was performed for MRgFUS.
[48]	Osteosarcoma: limb salvaging treatment by ultrasonographically guided high-intensity focused ultrasound	Osteosarcoma	Thermal ablation	7	Complete response in 3 patients, partial response in 3 patients. One patient had pulmonary metastasis 5 months post- HIFU. Median survival: 68 months Five-year survival rate: 71.4%
[49]	Noninvasive treatment of malignant bone tumors using high-intensity focused ultrasound	Osteosarcoma	Thermal ablation	25	100% of patients had significant pain relief 87.5% had complete pain relief *Primary* *bone tumors group:* Six (46.2%) complete response Five (38.4%) partial response One moderate response One progressive disease 84.6% total response rate *Metastatic bone tumors group*: Five (41.7%) complete response Four (33.3%) partial response One moderate response One stable disease One progressive disease 75.0% total response rate
[15]	Primary bone malignancy: effective treatment with high-intensity focused ultrasound ablation	Typical osteosarcoma, periosteal osteosarcoma, periosteal sarcoma, chondrosarcoma, Ewing sarcoma	Thermal ablation	Typical Osteosarcoma: 6 Periosteal Osteosarcoma: 1 Periosteal Sarcoma: 1 Chondrosarcoma: 10 Ewing Sarcoma: 3	Sixty-nine patients had complete ablation of their tumors. Eleven patients had >50% tumor ablation. Overall survival rates at 1, 2, 3, 4, and 5 yrs: 89.8%, 72.3%, 60.5%, 50.5%, and 50.5% Patients with stage IIb disease survival rates: 93.3%, 82.4%, 75.0%, 63.7%, and 63.7% Patients with stage III disease: 79.2%, 42.2%, 21.1%, 15.8%, and 15.8% Only five (7%) of the 69 patients who underwent complete ablation had local cancer recurrence. Forty adverse events were recorded.
[50]	High-intensity focused ultrasound (HIFU) is not indicated for treatment of primary bone sarcomas.	Osteosarcoma	Thermal ablation	N/A	*Commentary on [47] paper*: Authors argue that surgical remission is the most important prognostic factor for osteosarcomas, so surgery should not have been withheld. HIFU should not be advertised as a safe alternative to surgery unless it has the same rates of local control as surgery.
[51]	New clinical application of high-intensity focused ultrasound: local control of synovial sarcoma.	Spindle cell sarcoma	Thermal ablation	1	51-year-old male patient with recurrent synovial sarcoma (treated with lumpectomy and multiple cycles of chemotherapy) of left chest wall underwent 5 cycles of HIFU treatment, which completely ablated tumor. No adverse events reported.
[13]	High-intensity focused ultrasound: noninvasive treatment for local unresectable recurrence of osteosarcoma	Osteosarcoma	Thermal ablation	27	Two (7.4%) complete response Twelve (44.4%) partial response Nine (33.3%) stable disease Four (14.8%) progression Response rate: 51.8% Local disease control rate: 85.2%. Patients without pulmonary metastasis had better local disease control rate, longer local disease progression-free time, progression-free time, and overall survival time than patients with pulmonary metastasis.
[52]	Pediatric Sarcomas Are Targetable by MR-Guided High Intensity Focused Ultrasound (MR-HIFU): Anatomical Distribution and Radiological Characteristics	Pediatric sarcomas	Thermal ablation	121	Primary lesions: 64% targetable by MR-HIFU Majority of targetable tumors were osteosarcomas (31%) and Ewing sarcoma (21%) Metastatic tumors: 14% targetable Relapsed disease: 35% at least one targetable tumor Most metastases at diagnosis (79%) and lesions in recurrent disease (66%) were in the chest and not targetable 2/2 difficulties in ultrasound transmission and respiratory motion.
[16]	Significance of HIFU in local unresectable recurrence of soft tissue sarcoma, a single-center, respective, case series in China	Recurrent local, unresectable STS	Thermal ablation	Lipoblastoma: 8 Undifferentiated pleomorphic sarcoma: 7 Fibrosarcoma: 6 Chondrosarcoma: 4 Synovial sarcoma: 3 Leiomyosarcoma: 3 Aggressive fibromatosis: 2 Alveolar rhabdo- myosarcoma: 1 Clear cell sarcoma: 1 Primitive neuroectodermal tumor: 1	Zero complete response 47.3% partial response 33.3% had stable disease 19.4% had disease progression Twelve months after HIFU treatment: 38.9% partial response, 16.7% stable disease, 44.4% progression. Median LPFS, PFS, and OS were 13 months, 10 months, and 20 months, respectively. Twenty-seven patients had disease progression after 12-months, 16 of these patients had metastasis prior to secondary relapse of local recurrence, 9 had metastases after local secondary relapse, and 2 had simultaneous metastases and local secondary relapse. 33 patients reported pain prior to receiving HIFU; 9 achieved complete remission of pain, 16 achieved partial remission, and 8 reported no improvement in pain.
[53]	Portable ultrasound-guided high-intensity focused ultrasound with functions for safe and rapid ablation: prospective clinical trial for uterine fibroids-short-term and long-term results	Uterine leiomyoma	Thermal ablation	59	At 1-, 3-, and 5-months, fibroid volume shrinkage was 17.3%, 33.3%, and 45.1% At 3 month follow-up, 30.8% of patients reported >50% improvement in menorrhagia symptoms. Significant QOL improvement reported without changes in physical condition. Twenty-six patients satisfied with HIFU treatment and patient satisfaction was negatively correlated with residual tumor volume. Five patients underwent surgical myomectomy or hysterectomy, and one patient underwent hormonal intrauterine de-vice insertion due to recurrence of symptoms.
[5]	MRI features and clinical outcomes of unexpected uterine sarcomas in patients who underwent high-intensity focused ultrasound ablation for presumed uterine fibroids	Uterine sarcoma	Thermal ablation	17	Eleven patients with presumed uterine fibroids were diagnosed with uterine sarcoma prior to HIFU treatment. Six patients with presumed uterine fibroids were diagnosed with uterine sarcoma after treatment. There were no significant differences between histological type, margin of lesions, or enhancement of lesions on MRI that could explain the cause of misdiagnosis.
[54]	High-intensity focused ultrasound treatment as an alternative regimen for myxofibrosarcoma	Recurrent myxofibrosarcoma	Thermal ablation	1	After 5 cycles of low-power HIFU: Complete ablation of the tumor occurred. No tumor relapse was noted on serial MRIs during a 30 month follow-up period. No complications from HIFU treatment, possibly due to multiple, lower power treatment.
[14]	Managing spindle cell sarcoma with surgery and high-intensity focused ultrasound: A case report	Spindle cell sarcoma	Thermal ablation	1	After 5 cycles of HIFU ablation: Complete ablation of the tumor occurred. No complications from HIFU treatment reported.

**Table 3:** Current Clinical Trials.

Clinical Trial Name	clinicaltrials.gov ID	Status	Study Type	Study Location	Patient Population	Estimated Completion Date	Estimated Enrollment	Tumor Type(s)	Treatment Type	Study Overview
Focused Ultrasound to Promote Immune Responses for Undifferentiated Pleomorphic Sarcoma	NCT04123535	Recruiting	Interventional	San Francisco, USA	Adults	July 2025	20	Newly diagnosed or metastatic undifferentiated pleomorphic sarcomas	MRgFUS	**Primary outcome**: Evaluate rate and severity of adverse events from MRgFUS of undifferentiated pleomorphic sarcoma. **Secondary outcomes**: Measure immune responses to MRgFUS via serological analysis and multiplex immunohistochemistry assays of tumor specimens. Additionally, the immune responses to MRgFUS will be compared to the immune responses prior to receiving ultrasound in the same patients or to a comparison group of archived samples that did not undergo ultrasound.
HIFU Ablation of Soft Tissue Sarcoma	NCT05111964	Recruiting	Interventional	Oxford, UK	Adults	October 2025	12–16 with a minimum of 10	Soft tissue sarcomas and unresectable small symptomatic intra-abdominal desmoid tumors	High Intensity Focused Ultrasound (HIFU) ablation	**Primary outcome**: Measure safety and feasibility of HIFU ablation of soft tissue sarcomas and small symptomatic desmoid tumors. **Secondary outcome**: Radiological response using MRI and 18F-FDG PET will also be used to measure efficacy. Post-resection histology will also be completed to allow for histopathological correlation. **Tertiary outcome**: Exploration of immune response during HIFU ablation.

## Data Availability

No new data were generated or analyzed in support of this review.

## Abbreviations

The following abbreviations are used in this manuscript:

HIFU	High Intensity Focused Ultrasound
SDT	Sonodynamic Therapy
S180	Sarcoma 180 cell line
NPVR	Non-Perfused Volume Ratio
SCS	Spindle Cell Sarcoma
MRI-HIFU or MRgFUS	Magnetic Resonance Imaging-guided High Intensity Focused Ultrasound or Magnetic Resonance-guided Focused Ultrasound
HT	Hyperthermia
